# Favorable Short-term Outcome in Deceased Kidney Donor Transplantation

**Published:** 2012-05-01

**Authors:** M. H. Nourbala, M. R. Fatahi, B. Einollahi

**Affiliations:** Nephrology and Urology Research Center, Baqiyatallah University of Medical Sciences, Tehran, Iran

Dear Editor,

We read with great interest the article entitled “cadaver transplantation in recent era: is cadaveric graft survival similar to living kidney transplantation?” recently published in *IJOTM *by Simforoosh, *et al* [1]. The main message of this retrospective study is that one-year graft and patient survival rates in living donor kidney transplantation (LDKT) are similar to those of deceased donor kidney transplantation (DDKT) recipients.

It is of interest that authors showed that the short-term outcomes in DDKT were similar to LDKT [1]. We agree that the short-term outcome of renal transplantation has been improved in recent years, although it has been generally acknowledged that graft and patients survivals in LDKT grafts are superior to DDKT grafts. We evaluated the short-term outcomes of 121 adult deceased-donor recipients who underwent kidney transplantation at Baqiyatallah Transplant Center between 2008 and 2009 (unpublished data). One- and two-year graft survival rates were 94.0% and 86.8%, respectively. One- and two-year patient survival rates were also promising—97.4% and 91.9% respectively. Thus, our study also indicated a favorable improvement in the short-term graft and patient survivals of DDKT recipients. Mahdavi, *et al* [2], have also reported a good short-term outcome of DDKT grafts from Mashhad, Iran.

Simforoosh, *et al* [1], claimed that the main reason for obtaining such a good result in their DDKT patients was having a short cold ischemia time (period between harvesting and transplantation), *i.e.*, less than three hours. Since Iranian organ procurement is local now, cold ischemia time in our patients was also short which may be the key to have a favorable short-term outcome in our series too. The mean±SD cold ischemia time in our recipients was 3.16±0.83 (range: 1.5–4.7) hours which was relatively short. Cold ischemia time is an important risk factor for delayed graft function (DGF) and worsens the short-term transplant outcome [3]. It was shown that a reduction of cold ischemia time from 21.45 to 13.27 hours was associated with a significant decline in DGF rate from 34.7% to 20.7% [3].

Considering the favorable short-term outcomes in kidney transplants using deceased-donors, this practice should be encouraged in all transplant centers in Iran.
